# Association of auditory Charles Bonnet syndrome with increased blood flow in the nondominant Brodmann area 22

**DOI:** 10.1002/pcn5.92

**Published:** 2023-05-10

**Authors:** Hitoshi Sakimoto, Yuka Urata, Takanori Ishizuka, Hiroshi Kimotsuki, Motofumi Kasugai, Ryuji Fukuhara, Akira Sano, Masayuki Nakamura

**Affiliations:** ^1^ Department of Psychiatry Kagoshima University Graduate School of Medical and Dental Sciences Kagoshima Japan; ^2^ Department of Psychiatry Kagoshima City Hospital Kagoshima Japan; ^3^ Kagoshima Prefecture Mental Health and Welfare Center Kagoshima Japan; ^4^ Kagoshima University Kagoshima Japan

**Keywords:** auditory Charles Bonnet syndrome, Brodmann area 22, carbamazepine, musical hallucination

## Abstract

**Aim:**

Auditory Charles Bonnet syndrome (aCBS) is characterized by musical hallucinations (MHs) that accompany acquired hearing impairments. This hallucination is the acoustic perception of music, sounds, or songs in the absence of an outside stimulus, and it may be associated with hyperactivity of the superior temporal lobes. Some studies have reported the possibility of improving MH with antiepileptics. To elucidate in detail the brain regions responsible for aCBS, we analyzed the regions that changed functionally after treatment.

**Methods:**

Before and after treatment with carbamazepine (four cases), clonazepam (one case), and a hearing aid (one case), cerebral perfusion single‐photon emission computed tomography (SPECT) and the Auditory Hallucination Rating Scale (AHRS) were applied to six patients with hearing‐loss–associated MHs.

**Results:**

Cerebral blood flow analysis using SPECT revealed hyperperfusion in Brodmann area (BA) 22—the posterior region of the superior temporal gyrus—in the nondominant hemisphere in all six patients in the pretreatment phase. After treatment, the hyperperfusion region improved in all patients. The area percentages with hyperperfusion in the nondominant BA22 were strongly positively correlated with the AHRS score.

**Conclusion:**

The results suggest that aCBS, which was treatable with antiepileptics or hearing aids, was involved in hyperexcitement in BA22, and that MH strength was correlated with degree of excitement.

## Graphical abstract

To elucidate in detail the brain regions likely associated with auditory Charles Bonnet syndrome (aCBS), we analyzed brain regions that functionally changed after treatment. Cerebral blood flow analysis revealed hyperperfusion in Brodmann area 22, the posterior region of the superior temporal gyrus, of the nondominant hemisphere in patients with aCBS, which was treatable with antiepileptics or hearing aids.

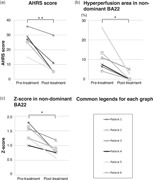

## INTRODUCTION

Charles Bonnet syndrome (CBS) is a clinical manifestation in which visually impaired people experience vivid visual hallucinations.^1^ Recently, musical hallucinations (MHs) observed in elderly people with hearing loss have been termed auditory CBS (aCBS).^2^ These hallucinations are a particular type of acoustic hallucination, and individuals with MHs perceive complex sound in the form of music in the absence of an acoustic stimulus. The condition is associated with known risk factors, such as brain disease, sensory impairment, and age.^3^ However, it is a rare and poorly understood clinical phenomenon, and the MH–auditory hallucination relationship has been poorly explored.^4^ Various medicines are used to treat the condition, including carbamazepine (CBZ).

Some studies have focused on the neuronal basis of aCBS and suggested that MH is associated with hyperactivity in the superior temporal gyrus (STG).^5^ Blood flow in the right STG increases during MHs, and the nondominant hemisphere is associated with this phenomenon.^3^, ^6^ The STG is composed of Brodmann areas (BAs) 22, 38, 41, and 42.

To identify the specific brain regions that are likely associated with aCBS, we analyzed pre‐/posttreatment changes in blood flow and the degree of MH in patients with aCBS.

## METHODS

### Subjects

Six Japanese patients with aCBS who consulted the Department of Psychiatry at Kagoshima University Hospital (Kagoshima, Japan) participated in this study. Written informed consent was obtained from all participants after the study had been approved by the hospital's institutional review board.

Patients 1–3 were analyzed retrospectively, while Patients 4–6 were enrolled in the study prior to their treatment for aCBS. Detailed patient profiles are given in Table 1. All patients with MH in this study experienced hearing loss. Each patient's clinical course is as follows.

**Table 1:** Detailed patient profiles.

Patient: age (years), sex	Patient 1: 63, male	Patient 2: 88, female	Patient 3: 90, female	Patient 4: 75, female	Patient 5: 88, male	Patient 6: 80, female
Handedness	Right	Right	Right	Left	Right	Right
Family history	HD in all brothers and father	–	–	HD in younger sister	–	–
Past history	Bil HD at 40sDEP at 59DM at 62	Right COM at 68Right HD at 68CI at 85Left HD at 87	Bil HD at 80sDM at 87	Left HD at 3Right HD at 60s	Bil HD at 60sHT	Bil HD at 75CRC at 77
Content of AH	African ethnic music of drum sounds	Japanese folk song involving multiple instruments and lyrics	Men's voice, Japanese folk song involving multiple instruments and lyrics	Men's voice, a melody of a hymn	Japanese folk song involving multiple instruments and lyrics, Palinacousis	Piano sound of Japanese song
Other symptoms	–	–	–	–	–	–
Hearing ability	Right: 71 dBLeft: 63 dB	Right: 105 dBLeft: 111 dB	Right: 105 dBLeft: 111 dB	Right: 69 dBLeft: 105 dB	Right: 50 dBLeft:60 dB	Right: 55 dBLeft: 58 dB

**Abbreviations:** AH, auditory hallucination; Bil, bilateral; CI, cerebral infarction; COM, chronic otitis media; CRC, colorectal cancer; dB, decibel; DEP, depression; DM, diabetes mellitus; HD, hearing difficulty; HT, hypertension.

Patient 1 was a 63‐year‐old right‐handed man who developed bilateral hearing loss in his 40s. He had a history of diabetes and hypertension but not of psychiatric illness. At age 59, he developed depression and claimed to hear African ethnic music. Clonazepam (0.5 mg) or aripiprazole (24 mg) did not improve his depressive symptoms or MH, whereas they were transiently relieved by antidepressants. Although his depressive symptoms did not recur, his MH relapsed. As a result of additional treatment with CBZ (100 mg/day), his MH disappeared.

Patient 2 was an 88‐year‐old right‐handed woman who developed right‐sided chronic otitis media with cholesteatoma at the age of 68 and completely lost hearing in the right ear. She had a history of hypertension and old cerebral infarction but not of psychiatric illness. At age 87, she also lost hearing in the left ear. Subsequently, she claimed to hear Japanese folk songs. This MH was persistent and became worse when she was in a quiet place. After treatment with CBZ (60 mg/day), her MH was relieved, but it relapsed after CBZ discontinuation. Her MH gradually transformed into interactive auditory hallucination. These symptoms were relieved after CBZ (60 mg/day) was resumed, although lamotrigine had no effect.

Patient 3 was a 90‐year‐old right‐handed woman who developed bilateral hearing loss in her 80s. She had a history of diabetes but not of psychiatric illness. At age 89, she complained of hearing music from the ceiling. At first, it was only the sound of a piano, and over time it came to be accompanied by lyrics. The melody of the MH was a folk song that she had heard before, and the singing voice was that of someone she knew. Antipsychotics had no effect. For a while, the MH gradually transformed into interactive hallucination. Treatment with CBZ (150 mg/day) reduced the frequency of the auditory hallucinations, but they did not disappear completely.

Patient 4 was a 75‐year‐old left‐handed woman with congenital hearing loss in her left ear. She also developed hearing loss in her right ear in her 60s. This was accompanied by the onset of an auditory hallucination of a Buddhist sutra, which gradually became MH. Her MH was relieved with CBZ treatment (400 mg/day).

Patient 5 was an 88‐year‐old right‐handed man who developed hearing loss when he was in his 60s. He had a history of hypertension but not of psychiatric illness. At age 87, he claimed to hear Japanese folk songs and tinnitus. He was treated with antidepressants, but his MH showed no improvement. With clonazepam (1 mg/day) administration, his MH improved.

Patient 6 was an 80‐year‐old right‐handed woman who had a history of colorectal cancer but not of psychiatric illness. She developed hearing loss around age 75. She started hearing the sound of a piano in the middle of the night at age 78. Sulpiride treatment was ineffective. Her auditory hallucinations improved with the use of hearing aids.

### Study protocol

We confirmed that the patients had no history of any disease that may have caused the psychotic symptoms of auditory hallucinations. Further, to ascertain that the patients had no severe cognitive impairment, we administered the Mini Mental State Examination (MMSE), head magnetic resonance imaging (MRI), and electroencephalography (EEG). We excluded any patients with a history of disease‐causing psychotic symptoms and/or severe cognitive dysfunction.

All patients underwent single‐photon emission computed tomography (SPECT) investigations and were examined using the Auditory Hallucination Rating Scale (AHRS) pre‐ and posttreatment. The AHRS measures frequency, reality, loudness, number of voices, length, attentional salience (how demanding of attention the voice is), and distress.^7^ Items are rated on a 5–10‐point scale, each with unique anchors. In addition, we conducted the Edinburgh Handedness Test to determine the patient's dominant hand. Patients 1–4 were administered CBZ. We performed functional MRI (fMRI) on Patient 4, who was left‐handed, to identify her dominant hemisphere, in conjunction with the shiritori game and the verb‐generation test.^8^, ^9^ Shiritori is a Japanese word game in which players take turns saying a word beginning with the last letter of the previous word. In the present study, we focused on the BA22 region of the nondominant hemisphere, which exhibited changes on SPECT before and after treatment for Patients 1–3.

### Regional cerebral blood flow study

We conducted SPECT investigations pre‐ and posttreatment to measure regional cerebral blood flow (rCBF). We performed the SPECT after an intravenous injection of 167 MBq of *N*‐isopropyl‐p‐(^123^I) iodoamphetamine using a gamma camera (GCA‐9300R; Canon) with a fan‐beam collimator, permitting a spatial resolution of 6.8 mm full‐width at half maximum. All SPECT image data were converted to a binary format.

Data analysis was performed with 3D‐SSP^10^ using iSSP freeware (Nihon Medi‐Physics). The rCBF for each 2.25 mm voxel was calculated by normalizing each voxel's activity to the global brain activity. The *Z*‐score [(mean−individual value)/standard deviation] of the images of each patient was calculated. Control datasets were constructed using the iSSP software from data for healthy volunteers (11 in the 60–64‐years age group and 16 in the 75–80‐years age group) at Chiba University (Chiba, Japan). Patients 1, 4, and 6 were compared with their respective age‐matched control groups. Patients 2, 3, and 5, who were over 80 years old, were compared with the 75–80‐years age control group.

In this study, we analyzed cerebral blood flow using the stereotactic extraction estimation (SEE) method, which describes the orientation and expansion of regions of accumulation by classifying stereotactic brain coordinates according to the brain's anatomical structure.^11^ This method uses a five‐level classification of brain regions as used by the Talairach Daemon software. We identified the BA‐level (Level 5) classification of areas exhibiting hyperperfusion using the SEE method, and we calculated the percentage of each area that exhibited hyperperfusion (voxels with *Z* ≥ 2).^12^ We then compared the mean Z‐score for BA22 and the percentage of the area of BA22 that had hyperperfusion between our pre‐ and posttreatment findings. The mean *Z*‐score provided an overall measure of blood flow in BA22, while the percentage of the area with hyperperfusion provided information about the spatial distribution of blood flow within BA22.

### Statistical analysis

Paired Student's *t*‐tests were performed to compare pre‐ and posttreatment findings. Spearman's rank correlation coefficient tests were performed to determine correlations between the AHRS and *Z*‐score, AHRS and degree of hearing loss, and hyperperfusion area and degree of hearing loss at the pretreatment stage. A *p*‐value < 0.05 was considered to indicate a statistically significant difference in the paired Student's *t*‐tests. The probabilities of significance were determined using the Bonferroni correction to address the issue of multiple comparisons of bilateral BA22s. The effect sizes were calculated using G*Power 3.

## RESULTS

Table 2 presents the detailed results of examinations, excluding those of the SPECT investigations. The MMSE scores were not indicative of severe cognitive decline in any patient,^13^ head MRIs revealed neither severe cerebral infarctions nor tumors, and the EEG revealed no abnormalities. Hematological findings showed elevated HbA1c in Patients 1 and 2. Although Patient 2 was positive for antinuclear antibodies, no increased inflammatory response or clear autoimmune disease was identified. Because only Patient 4 was left‐handed, we conducted an fMRI in conjunction with the shiritori game and the verb‐generation test, which revealed that language production in this patient was right‐lateralized.^8^, ^9^ Patients 1–4 were administered CBZ.

**Table 2:** Detailed results of examinations.

Patients	Patient 1	Patient 2	Patient 3	Patient 4	Patient 5	Patient 6
Hematological findings	HbA1c 6.2%, HCV antibody (+)	Antinuclear antibody, (+)Centromere, anticytoplasmic	HbA1c 6.5%	Not applicable	Not applicable	Not applicable
Head MRI	IC (+)	IC (+), Strong atrophy (+)	IC (+), Strong atrophy (+)	IC (+)	IC (+), Strong atrophy (+)	IC (+), Strong atrophy (+)
MMSE (Failing items)	26/30(Attention, Calculation, and Recall)	30/30	30/30	27/30(Orientation and Recall)	28/30(Attention and Calculation)	25/30(Attention, Calculation, and Recall)
EEG	No abnormality	No abnormality	No abnormality	No abnormality	No abnormality	No abnormality

**Abbreviations:** HCV, hepatitis C virus; IC, ischemic change; MMSE, Mini Mental State Examination.

### Comparison between pre‐ and posttreatment AHRS scores

**Figure 1:**
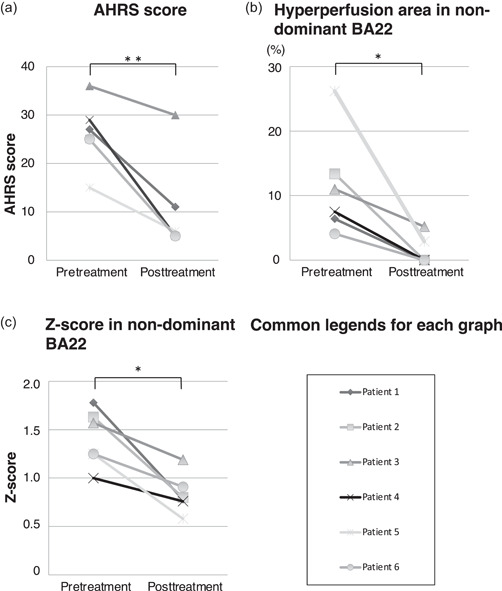
Comparison of magnitude of auditory musical hallucinations (MHs), area with hyperperfusion in terms of the regional cerebral blood flow (rCBF) in Brodmann area (BA) 22, and *Z*‐scores for rCBF in BA22 between pre‐ and posttreatment findings. (a) Pre‐ and posttreatment Auditory Hallucination Rating Scale (AHRS) scores. The scores were significantly reduced posttreatment (*p* = 0.003, paired *t*‐test, effect size 1.60). (b) The percentage of the area with hyperperfusion (*p* = 0.0187, paired *t*‐test, effect size 4.55), and (c) *Z*‐scores for rCBF (*p* = 0.0055, paired *t*‐test, effect size 2.84) in the nondominant BA22 decreased significantly posttreatment. *p < 0.05, **p < 0.01.

Figure 1a shows the pre‐ and posttreatment AHRS scores. In all patients, MH improved significantly posttreatment (*p* = 0.003, paired *t*‐test, effect size 1.60). Patients 1–4 were treated with CBZ, which was effective in all cases, as it led to a reduction in AHRS scores (*p* = 0.031, paired *t*‐test, effect size 1.86, data not shown).

### Changes in rCBF in the nondominant BA22

The rCBF was significantly lower posttreatment than pretreatment. We compared rCBF in the nondominant BA22 between pre‐ and posttreatment measurements (Figure 1b,c). The mean percentage of the area of BA22 showing hyperperfusion decreased significantly from pre‐ to posttreatment: 11.43 ± 7.27% and 1.35 ± 2.02%, respectively (*p* = 0.037, paired *t*‐test, effect size 4.55; Figure 1b). In all patients, the *Z*‐score in BA22 decreased posttreatment (*p* = 0.011, paired *t*‐test, effect size 2.84; Figure 1c).

### Correlation between hyperperfusion in the nondominant BA22, AHRS scores, and degree of hearing loss in the pretreatment condition

**Figure 2:**
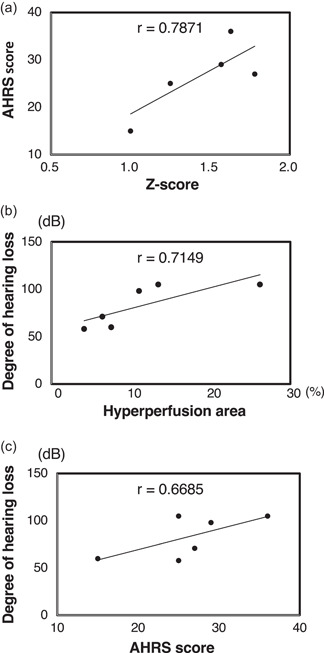
Correlation among the percentage of the area with hyperperfusion in the nondominant Brodmann area (BA) 22, the strength of musical hallucination (MH), and degree of hearing loss. (a) Patients 5 and 6 had the same values (*Z*‐scores of 1.25 and Auditory Hallucination Rating Scale [AHRS] scores of 25). The *Z*‐scores for regional cerebral blood flow (rCBF) in the nondominant BA22 were correlated with the AHRS scores (*r* = 0.7871, *p* = 0.0023). (b) The percentage of the area with hyperperfusion was correlated with the degree of hearing loss (*r* = 0.7149, *p* = 0.0089). (c) The AHRS scores were correlated with the degree of hearing loss (*r* = 0.6685, *p* = 0.0174).

In the pretreatment condition, correlations between the *Z*‐scores for rCBF in the nondominant BA22, AHRS scores, area percentages with hyperperfusion, and degree of hearing loss were analyzed. The *Z*‐scores for rCBF in the nondominant BA22 were strongly positively correlated with AHRS scores (*r* = 0.7871, *p* = 0.0023; Figure 2a). The area percentages with hyperperfusion in the nondominant BA22 were strongly positively correlated with the degree of hearing loss (*r* = 0.7149, *p* = 0.0089; Figure 2b). Degree of hearing loss was moderately strongly positively correlated with AHRS scores (*r* = 0.6685, *p* = 0.0174; Figure 2c).

## DISCUSSION

### Treatment for MH in aCBS

MH in the elderly occurs predominantly in females with hearing impairments. Various treatment options are available for MH, such as pharmacotherapy with antipsychotics, antiepileptics, anxiolytics, and acetylcholinesterase inhibitors. The condition is best treated by directing the intervention at the etiological mechanism underlying its mediation.^14^

The patients in our study—all of whom were elderly and had hearing loss, no previous psychiatric comorbidity, and no cognitive impairment—had MH as their major psychiatric symptom. This symptom in these patients was assumed to be caused by aCBS due to hearing loss. Patients 5 and 6 were not treated with CBZ. In Patients 1–4, CBZ was effective for treating MH with hyperperfusion in the nondominant BA22, while in Patients 1, 2, 3, 5, and 6, antidepressants, lamotrigine, antipsychotics, antidepressants, and sulpiride were ineffective, respectively. Treatment with CBZ induces a perceptual change in pitch,^15^ suggesting that it affects music perception, which may explain its effectiveness for treating MH in aCBS. MHs and the attendant pathology can be improved by stimulating auditory perception.

In the present study, one patient responded well to clonazepam and another did not. Because of the limited number of patients in this study, further investigation with more patients is required to determine the efficacy of clonazepam.

In Patient 6, her MH and hyperperfusion in BA22 improved with the use of hearing aids.^14^ Although this finding was also limited to only one patient, this result suggests that if some residual auditory perception remains, MH and its pathology may be improved by stimulating auditory perception.

### Relationship between hyperneuronal activity in the nondominant BA22 and MH in aCBS

Although an association between MH and hyperperfusion in the STG has been reported,^16^, ^17^ in the present study we analyzed rCBF in BA22, a more specific region of the STG. Hyperperfusion in the nondominant BA22 during MH was observed in all patients, supporting previous studies reporting STG hyperperfusion in the nondominant hemisphere. The AHRS score was correlated with the *Z*‐score for rCBF in the nondominant BA22, but not with the percentage of the area with hyperperfusion (*r* = 0.1811, *p* = 0.5733, data not shown). This suggests that the strength of the MH was correlated with the degree of hyperperfusion in the nondominant BA22. In addition, patients with a greater extent of hyperperfusion in BA22 tended to experience more complex MHs, involving multiple instruments and lyrics (Patients 2, 3, and 5; Figure 1b; Table 1). This finding suggests that the spatial distribution of hyperperfusion, rather than the overall level of blood flow, may be more closely related to the type of MHs experienced by patients with aCBS. This hyperperfusion improved posttreatment in all patients. These results further support the conclusion that hyperneuronal activity in the nondominant BA22 is a crucial aspect of the pathogenesis of MH in aCBS.

### Hearing loss and MH

MHs are more common in the elderly, and background factors, such as hearing loss, psychiatric disorders, cerebrovascular disease, brain tumor, diffuse cerebral atrophy, epilepsy, and medication have been reported to be associated with MH in this demographic. In such cases, MH is most commonly seen in patients with moderate or severe acquired deafness. It is therefore thought to constitute self‐activation of music perception, which may indicate aCBS pathophysiology.^2^, ^3^

In the present study, we found that the degree of hearing loss was correlated with MH strength. This suggests that as the degree of auditory deprivation increases, self‐activation of music perception becomes more likely. Patients 2–4 not only perceived melody but also experienced verbal auditory hallucinations. Compared with Patients 1, 5, and 6, who experienced only MHs, Patients 2–4 exhibited a larger area with hyperperfusion in the STG of the dominant hemisphere (*Z* > 0, hyperperfusion area: 61.6% vs 18.6%). These results suggest that the Wernicke's area, which is located in the posterior part of the STG in the dominant hemisphere, is associated with the hearing of hallucinatory voices accompanied by music.

The MH in Patient 1 appeared along with depression following the development of hearing loss. Berrios et al. reported that depression was identified in 26% of patients with MH, and that this depression may have been associated with the MHs.^2^ However, the pathophysiological relationship between MH and depression is unclear. Several studies on MH have revealed hyperactivity in the prefrontal cortex, cingulate, striatum, and basal ganglia, all of which are involved in the processing and production of speech and in emotional processing.^18^, ^19^ Although there is as yet no evidence for this, depression may trigger an imbalance in these areas, leading to a lower threshold for auditory musical perception, which may result in MH occurring as a result of neural activity heightened by hearing loss.

### Limitations

Since the number of patients in this case‐series study was limited, more cases and further confirmatory studies are needed. Ideally, all participants should have undergone an fMRI scan to identify their dominant hemisphere, since the dominant hemisphere of 4% of right‐handers is the right.^20^ However, in the present study, we performed the fMRI scan only for the one left‐handed patient with aCBS (Patient 4).

### Conclusion

The MH in aCBS was strongly positively correlated with blood flow in the nondominant BA22, both in terms of the strength of MH before treatment and in the pre‐/posttreatment comparison of MHs, suggesting that neuronal overactivity in the nondominant BA22 is involved in the pathogenesis of MH in aCBS. Although the number of patients in this study was limited, the effect size was large and may thus usefully reflect the pathophysiology of MH in aCBS.

As described by Coebergh et al., MH is best treated by directing intervention at the etiological mechanism responsible for its mediation.^14^ Treatment with CBZ was effective in at least four of the six patients, so this may be considered a treatment strategy for MH in aCBS, in addition to the use of hearing aids as a nonpharmacological treatment strategy for individuals with aCBS and residual auditory perception.

## AUTHOR CONTRIBUTIONS

Hitoshi Sakimoto, Akira Sano, and Masayuki Nakamura designed this study. Hitoshi Sakimoto, Akira Sano, and Masayuki Nakamura were responsible for the field investigation. Hitoshi Sakimoto performed the statistical analyses. Hitoshi Sakimoto and Masayuki Nakamura wrote the manuscript. Hitoshi Sakimoto, Yuka Urata, Takanori Ishizuka, Hiroshi Kimotsuki, Motofumi Kasugai, Akira Sano, and Masayuki Nakamura were directly or indirectly involved in the patients' treatment and in providing their clinical histories. Masayuki Nakamura, Ryuji Fukuhara, and Akira Sano reviewed and edited the manuscript. All authors approved the final version of the manuscript that was submitted.

## CONFLICT OF INTEREST STATEMENT

Masayuki Nakamura received an honorarium from Takeda Pharmaceutical Company Limited. Other authors declare no conflicts of interest.

## ETHICS APPROVAL STATEMENT

This study was approved by the Ethics Review Committee of Kagoshima University Hospital (No. 25‐106).

## PATIENT CONSENT STATEMENT

Written informed consent was obtained from all participants.

## CLINICAL TRIAL REGISTRATION

N/A

## Data Availability

The datasets generated and/or analyzed during the current study are available from the corresponding author on reasonable request.
